# Effects of the Dietary Supplement 5α‐Hydroxy‐Laxogenin in the Orchiectomized Rat Model

**DOI:** 10.1002/dta.3881

**Published:** 2025-03-04

**Authors:** Daniel Derwand, Oliver Zierau, Clemens Alexander Wolf, Gerhard Wolber, Annekathrin Martina Keiler

**Affiliations:** ^1^ Institute of Doping Analysis and Sports Biochemistry Dresden Kreischa Germany; ^2^ Faculty of Biology, Environmental Monitoring & Endocrinology Technische Universität Dresden Dresden Germany; ^3^ Molecular Design Lab, Institute of Pharmacy, Department of Biology, Chemistry and Pharmacy Freie Universität Berlin Berlin Germany

**Keywords:** doping, Hershberger assay, molecular modelling

## Abstract

Dietary supplements used by recreational and elite athletes for performance enhancement might contain undeclared, unlawfully added ingredients. One of those ingredients is 5α‐hydroxy‐laxogenin, which is sold in dietary supplements marketed as a natural compound with anabolic effects. It has been shown that 5α‐hydroxy‐laxogenin is not naturally occurring, but rather of synthetic origin. Previously, we observed that 5α‐hydroxy‐laxogenin can bind to and activate the androgen receptor in a cell‐based bioassay. To investigate its androgenic potential in vivo, we treated orchiectomized rats with three different dosages of 5α‐hydroxy‐laxogenin for 2 weeks. Effects were neither observed on the wet weights of the androgen target tissues prostate, seminal vesicle or penis nor on the wet weights of the anabolic target tissue *musculus levator ani* or on skeletal hindlimb muscles. Au contraire, significantly higher atrophy was seen for some of the target tissues in the animals treated with the highest 5α‐hydroxy‐laxogenin dosage (36 mg/kg bw). While in silico docking supports the androgen receptor binding previously observed in vitro, we observed neither androgenic nor anabolic effects of 5α‐hydroxy‐laxogenin in vivo in castrated male rats.

## Graphical abstract

The dietary supplement ingredient 5α‐hydroxy‐laxogenin was investigated in the castrated male rat for potential androgenic and anabolic effects. Moreover, androgen receptor binding of 5α‐hydroxy‐laxogenin was modelled by in silico docking. While molecular modelling supports androgen receptor binding, we observed neither androgenic nor anabolic effects in the castrated rat.

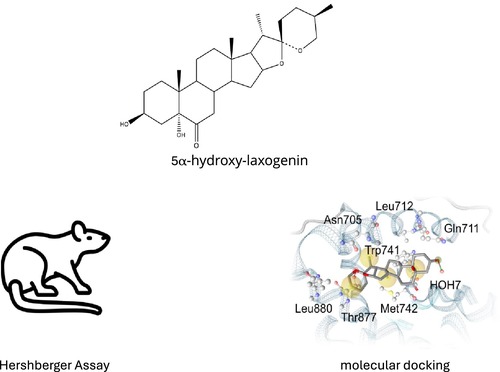

## 1 Introduction

Dietary supplements used by recreational and elite athletes for performance enhancement may contain undeclared, illegally added ingredients. These include pharmaceuticals (e.g. diuretics, stimulants and androgens) as well as unapproved substances, botanical extracts or animal‐derived traditional medicine formulations [1, 2, 3]. Regarding elite sport, the World Anti‐Doping Agency (WADA) publishes annually the List of Prohibited Substances and Methods [4]. The intake of dietary supplements contaminated with prohibited substances according to the Prohibited List can result in positive doping tests due to unintentional exposure [5]. The US Food and Drug Administration (FDA) publishes a directory with illegally added ingredients found in dietary supplements to warn consumers [6]. One of these ingredients is 5α‐hydroxy‐laxogenin, which is sold in dietary supplements marketed as a natural compound with anabolic effects. Two studies analysed several dietary supplements for the presence of 5α‐hydroxy‐laxogenin and other, unlabelled ingredients [7, 8]. Moreover, Avula et al. proved 5α‐hydroxy‐laxogenin's synthetic origin; hence, it is not of natural, plant‐derived origin [7]. Despite its labelling as unlawful ingredient of dietary supplements, which might further contain other nonlabelled performance enhancing drugs according to the FDA and a related warning by the US Anti‐Doping Agency (USADA) [9], 5α‐hydroxy‐laxogenin is not prohibited by WADA. Due to lack of scientific evidence of performance‐enhancing properties or potential health risks to athletes, the requirements for a potential prohibition of 5α‐hydroxy‐laxogenin are not fulfilled [10]. Recently, we demonstrated the androgen receptor (AR) activation by 5α‐hydroxy‐laxogenin in an in vitro bioassay using human PC3 cells [11]. However, there is no evidence that 5α‐hydroxy‐laxogenin has androgenic or anabolic potential in vivo. A preclinical bioassay to test compounds for androgenic properties is the Hershberger assay in castrated male rats according to the Organisation for Economic Co‐operation and Development (OECD) [12]. Using a slightly modified Hershberger assay, we treated male castrated rats with three different doses of 5α‐hydroxy‐laxogenin for a period of 2 weeks. Androgenic and anabolic target tissues were analysed.

## 2 Materials and Methods

### 2.1 Chemicals and Reagents

5α‐Hydroxy‐laxogenin (purity ≥ 98%) was purchased from ChemScene (Monmouth Junction, New Jersey, United States). Castor oil, dimethyl sulfoxide (DMSO), EDTA (98%) and testosterone (purity 99%) were purchased from Sigma–Aldrich (St. Louis, Missouri, United States). Morphisto GmbH (Offenbach am Main, Germany) supplied the Masson Goldner Trichrome staining kit. Mayer's hematoxylin, ROTI Histol, ROTI Histokitt, Schiff's reagent, paraformaldehyde (DAC, pure), periodic acid (99%) and Tris base were purchased from Carl Roth (Karlsruhe, Germany). Thermo Fisher Scientific (Waltham, Massachusetts, United States) supplied RNaseZap, TRIzol and Superfrost Plus adhesion microscope slides. Millipore (Burlington, Massachusetts, United States) supplied Millipore IHC Select HRP/DAB Kit. Sodium metabisulfite (98%) was purchased from Grüssing GmbH (Filsum, Germany). Teklad Global Diets 2016 (Teklad Laboratory Animal Diets Lafayette, Indiana, United States). Ketamine hydrochloride and xylazine (Xylariem) were purchased from Pharma Partner (Hamburg, Germany).

### 2.2 Animal Experiment

Male 4‐week‐old LEW/OrlRj rats (Janvier Labs, Genest‐Saint‐Isle, France) were housed under controlled conditions (20°C ± 1°C, 40%–70% relative humidity, 12:12‐h light dark cycles) in groups of three to four individuals with water and food access ad libitum. After 3 weeks of habituation, the animals underwent orchiectomy under a ketamine–xylazine anaesthesia combined with an intra‐ and postoperative pain therapy for three consecutive days (5 mg/kg bw carprofen s.c. administered) and followed by a 3‐week recovery. Subsequently, the animals were allocated to five different treatment groups: vehicle treatment group (*n* = 8), testosterone treatment (1 mg/kg body weight, *n* = 8) and three 5α‐hydroxy‐laxogenin dosage regimes (1 mg/kg bw, *n* = 8; 12 mg/kg bw, *n* = 9; and 36 mg/kg bw, *n* = 9). All test compounds were dissolved in DMSO and castor oil; animals received a daily s.c. injection (200 μL) for 2 weeks. The vehicle treatment group received DMSO and castor oil solely as solvent control.

At necropsy, animals were sacrificed 24 h after the last injections using CO_2_ inhalation after a light O_2_/CO_2_ anaesthesia. After tissue dissection and weighing, the tissue samples were either fixed in 4% paraformaldehyde for 48 h at 4°C prior to paraffin embedding or frozen in liquid N_2_. Animal handling and experimental procedure were in accordance with the ethical standards of the declaration from Helsinki from 1964, the 3R Principle and the German federal animal welfare law and were approved and carried out according to the Institutional Animal Care and Use Committee and official Governmental Animal Experimentation Commission (Approval Number 25‐5131/496/40).

### 2.3 Histological Analysis

Paraffin‐embedded liver, *M. soleus* and ventral prostate samples were cut in 3‐μm‐thick sections (Leica RM 2065, Leica Biosystems, Nußloch, Germany), transferred to microscope slides and dried overnight at 40°C. Very brittle tissue (already trimmed blocks) was incubated with 0.1% Tween 20 in H_2_O‐soaked tissues for 15–30 min before sectioning. Before staining, tissue sections were dewaxed and rehydrated. Liver sections were stained with Masson Goldner Trichrome according to the manufacturer's recommendations. Differing from that, the Orange G incubation was performed for 25 min. For each treatment group, liver sections from five rats were analysed. Skeletal muscle (*M. soleus*) sections were stained using periodic acid–Schiff staining.

Prostate sections were incubated overnight at 60°C in 10 mM Tris‐EDTA for antigen retrieval. The immunostaining was carried out with the Millipore IHC Select HRP/DAB kit and an anti‐Ki67 primary antibody (rabbit monoclonal anti‐Ki67 antibody [SP6], ab16667, Abcam, Lot No. GR3313195). Staining was performed according to the manufacturer's specifications except for the following steps: After suppression of endogenous peroxidases with 3% H_2_O_2_ for 10 min, 20% bovine serum albumin (BSA) in 1x TBS was used for blocking for 1 h at ambient temperature followed by incubation with the primary antibody for 1 h at ambient temperature (1:100 dilution in 20% BSA in 1x TBS). The counterstaining was carried out with Mayer's hematoxylin for 8 min and bluing under running, hand warm tap water (10 min.). For isotope control, we used the monoclonal rabbit IgG‐antibody (EPR25A‐Isotype Control, ab172730, Abcam, Lot No. 1011223−4). For each treatment group, seven animals were analysed. For each animal, random images (200× magnification) of the prostate sections were taken and at least 200 nuclei were counted to determine the percentage of Ki67 positive nuclei. For microscopy, an Axiolab with Zen2.0 (Carl Zeiss AG, Jena, Germany) was used. The evaluation of microscopic pictures was carried out with Fiji/ImageJ (Freeware, Wayne Rasband).

### 2.4 Gene Expression Analysis

RNA was isolated from frozen *M*. *soleus* samples using TRIzol (Thermo Fisher Scientific). The RNA concentration was determined by NanoPhotometer NP80 (Implen, München, Germany). Three thousand nanograms of RNA was treated with DNase I for 1.5 h at 25°C followed by enzyme inactivation by adding 1 μL 25 mM EDTA and a 5‐min incubation at 80°C. RNA was reverse transcribed into cDNA using the High‐Capacity cDNA Synthesis Kit (Thermo Fisher Scientific, Waltham, United States) according to the manufacturer's protocol. QPCR was performed on QuantStudio 5 Real‐Time PCR system with PowerUp SYBR Green Master Mix (Thermo Fisher Scientific, Waltham, United States) using the different primer mixes according to Table 1. The end volume was 10 μL per reaction. Relative expression levels were determined by ∆∆Ct analysis, normalizing to the two housekeeping genes Rps18 and 1A and in relation to the expression levels in the control group (set to 1).

**TABLE 1:** Primer sequences used for quantitative real‐time PCR.

	Primer sequence	Primer mix concentration (μM)
*Androgen receptor (AR)*	5′‐TGT GCC GGA CAT GAC AAC AA‐3′ 5′‐ACT TGT GCA TGC GAT ACT CAT T‐3′	0.4
*Myogenin*	5′‐GAC CCT ACA GGT GCC CAC AA‐3′ 5′‐ACA TAT CCT CCA CCG TGA TGC T‐3′	0.2
*MyoD1*	5′‐GCG ACA AGC CGA TGA CTT CTA T‐3′ 5′‐GGT CCA GGT CCT CAA AAA AGC‐3′	0.4
*RPS18* (reference gene)	5′‐CGT GAA GGA TGG GAA GTA TAG C‐3′ 5′‐TAT TAA CAG CAA AGG CCC AAA G‐3′	0.5
*1A* (reference gene)	5′‐TGA GCA GGA ATA GTA GGG ACA GC‐3′ 5′‐GAG TAG AAA TGA TGG AGG AAG CA‐3′	0.5

### 2.5 Protein Preparation and Molecular Docking

There are 81 crystal structures of human AR available in the Protein Data Bank (PDB) [13]. The binding site of AR is liable to conformational changes introduced by the ligand. Thus, in order to select the AR conformation most suitable for modelling the binding of 5α‐hydroxy‐laxogenin, we searched for the crystal structure harbouring a ligand with the highest shape and interaction motif similarity to 5α‐hydroxy‐laxogenin. Only x‐ray 3D structures of the wild‐type receptor containing a cocrystallized ligand were considered. The cocrystallized ligands extracted from their protein structures were subjected to 3D conformation sampling using OMEGA v. 4.1.2.0 (OpenEye Scientific Software, Santa Fe, New Mexico, United States) [14] with ‘strict stereo’ disabled so as to allow for the handling of stereochemically unspecified ligands and a maximum of 200 conformations per ligand. All other parameters were kept at default settings. Subsequently, ROCS v. 3.4.3.0. (OpenEye Scientific Software, Santa Fe, New Mexico) [15] was used to query the resulting database for the ligand bearing the highest shape and interaction similarity to 5α‐hydroxy‐laxogenin measured by TanimotoCombo (shape and colour) score [15]. The ligand with the highest similarity to 5α‐hydroxy‐laxogenin turned out to be EM‐5744 (PDB Code 2PNU [16]), a rationally designed steroid analogue. Consequently, the crystal structure featuring EM‐5744 with an atomistic resolution of 1.65 Å served as input template for molecular docking experiments. The protein structure (PDB Entry 2PNU [16]) was prepared in MOE v. 2020.0901 [17] including protonation at pH 7 with Protonate3D [18] and 300 K and removal of all water molecules apart from the water molecule HOH7 located between Arg752, Met745 and EM‐5744 3‐keto group. This water molecule is suspected to be relevant for ligand binding, because it forms a hydrogen bond network between EM‐5744 3‐keto group and residues Arg752 and Met745 in the crystal structure (PDB Entry 2PNU [16]). A possible role for a water molecule located between Met745 and Arg752 and the ligand in AR has been discussed previously and is found in AR structures repeatedly [19]. Molecular docking of 5α‐hydroxy‐laxogenin was conducted using GOLD v. 5.8.1 [20]. The C6 atom of the steroid scaffold of EM‐5744 was used as the centre of a docking sphere of 20 Å, as this atom approximates the centre of the AR binding site. Search efficiency of 100% was used and the genetic algorithm (GA) was set to run 50 times. The resulting poses were assessed employing the GoldScore P450 scoring function and subsequently energetically minimized in the MMFF94 force field [21] implemented in LigandScout v. 4.4.3 [22] within the AR environment. The best pose was chosen based on resemblance to the cocrystallized ligand in terms of both shape and interaction pattern in LigandScout.

### 2.6 Statistical Analysis

Data are shown as mean ± standard deviation. The statistical evaluation of the acquired data was performed using OriginLab2021 (OriginLab, Northampton, Massachusetts, United States) applying the Shapiro–Wilk test for normal distribution, Grubbs test for outliers and one‐way ANOVA followed by Bonferroni post hoc test for normally distributed data or Mann–Whitney test for nonnormally distributed data.

## 3 Results and Discussion

### 3.1 In Silico Docking of AR Binding

**FIGURE 1:**
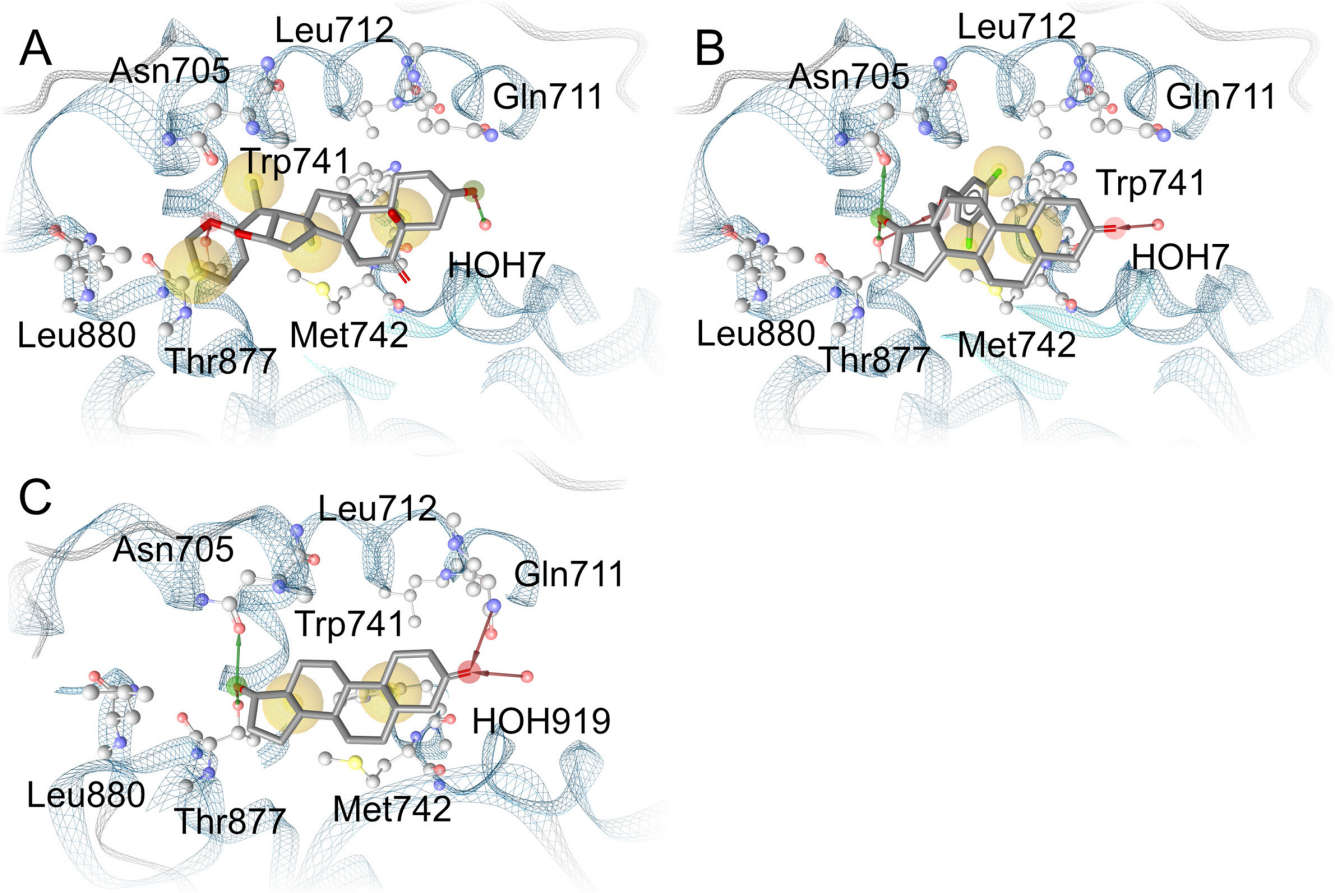
(A) Binding hypothesis of 5α‐hydroxy‐laxogenin in human AR. (B) Cocrystallized ligand EM‐5744 in human AR (PDB Code 2PNU) [16]. (C) Cocrystallized ligand DHT (PDB Code 3L3X [23]). Green arrows indicate hydrogen bonds donors, red arrows indicate hydrogen bond acceptors, and yellow spheres indicate hydrophobic contacts.

The most suitable x‐ray structure of AR (PDB ID: 2PNU, cocrystallized with ligand EM‐5744 [16]) was chosen according to three‐dimensional ligand similarity (see the Materials and Methods section for details) for docking experiments of 5α‐hydroxy‐laxogenin in human AR. The binding pose resulting from our docking experiments (Figure 1A) exhibits a similar shape and interaction pattern to both EM‐5744 cocrystallized in the crystal structure used (PDB Code 2PNU; Figure 1B) and also endogenous ligand DHT cocrystallized in PDB Entry 3L3X [23] (Figure 1C). Our binding hypothesis indicates that the 3‐hydroxyl group of 5α‐hydroxy‐laxogenin forms hydrogen bonds to conserved water HOH7. A water molecule in this position is involved in a hydrogen bond network facilitating the binding of both EM‐5744 [16] and DHT [23] in their respective crystal structures. This water molecule is indicated to play a role in the binding of steroids to the AR via the 3‐keto group [19]. Additionally, the ether oxygen in the tetrahydropyran ring of 5α‐hydroxy‐laxogenin acts as hydrogen bond acceptor to the Thr877 hydroxyl group. EM‐5744 also engages in hydrogen bonding with HOH7 via its 3‐keto group, as does DHT (HOH919 in 3L3X corresponds to HOH7 in 2PNU). The Thr877 hydroxyl group forms a hydrogen bond with the 17‐hydroxy group in both DHT and EM‐5744. In addition, both EM‐5744 and DHT form a hydrogen bond to Asn705 delta oxygen with the ligand's 17‐hydroxyl group as hydrogen bond donor. In contrast, 5α‐hydroxy‐laxogenin is unable to act as hydrogen bond donor to Asn705, as its ether oxygen moieties can only act as hydrogen bond acceptor. Furthermore, EM‐5744 ether oxygen in the 13‐ethoxy moiety forms an additional hydrogen bond with the Thr877 hydroxyl group and DHT 3‐keto functionality forms a hydrogen bond to Gln711 epsilon nitrogen, both as acceptor. The comparable conformations of 5α‐hydroxy‐laxogenin, EM‐5744 and DHT are complemented by a coat of lipophilic contacts. Residues involved include Trp741 and Met742 for all three ligands and additionally Leu880 for 5α‐hydroxy‐laxogenin and Leu712 for EM‐5744.

### 3.2 Effects on Androgenic and Anabolic Target Tissues

**FIGURE 2:**
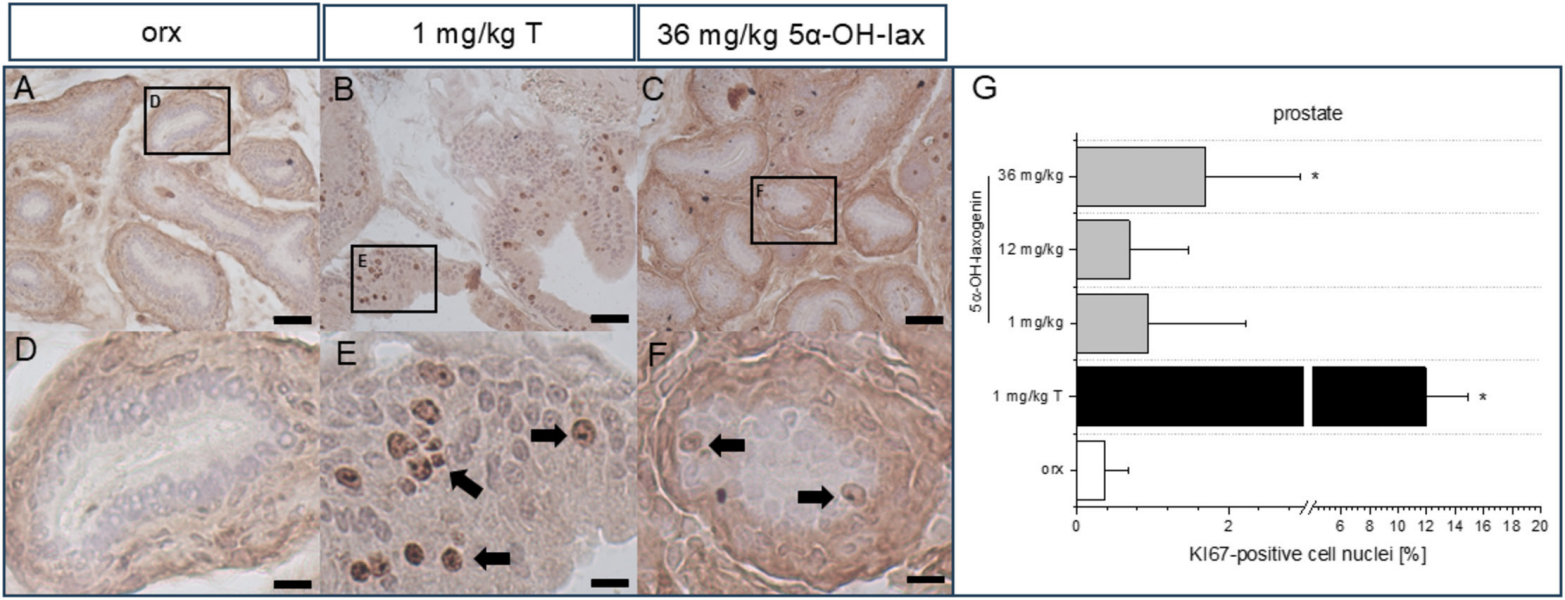
Light microscopy of KI67 prostate staining in (A, D) the control group (orx), (B, E) the testosterone‐treated group (1 mg/kg T) and (C, F) the 36 mg/kg 5α‐hydroxy‐laxogenin‐treated group (36 mg/kg 5α‐OH‐lax). (G) KI67‐positive cell nuclei (200 nuclei/animal for 7 animals/group), *p ≤ 0.05 versus orx control group. → points to Ki67‐positive cell nuclei. Scale bar = 50 μm (A–C), scale bar = 20 μm (D–F).

The orchiectomy‐induced atrophy of the androgenic target tissues prostate, seminal vesicle and the penis was prevented by daily treatment with 1 mg/kg testosterone (Table 2). In contrast, the two lower dosages of 5α‐hydroxy‐laxogenin had no significant impact on the wet weights of the androgen‐dependent target tissues compared to the control group. In response to the highest 5α‐hydroxy‐laxogenin dose, the relative wet weight of the glans penis was significantly lower compared to the orchiectomized control animals (0.298 ± 0.037 g/kg vs. 0.412 ± 0.077 g/kg, Table 2). Immunohistochemical staining of the ventral prostate (prostate sections are shown in Figure 2A–F) revealed a significant increase of KI67‐positive cell nuclei in the testosterone‐treated animals (11.9% ± 3.0% vs. 0.366% ± 0.32% in the orx control group, Figure 2G).

**TABLE 2:** Wet weights of target tissues relative to the bodyweight at necropsy. Animals were treated for 2 weeks by daily subcutaneous injections.

	orx	1 mg/kg testosterone	5α‐hydroxy‐laxogenin		
			1 mg/kg	12 mg/kg	36 mg/kg
Prostate (g/kg bw)	0.482 ± 0.058	1.499 ± 0.164*	0.496 ± 0.100	0.459 ± 0.093	0.509 ± 0.182
Seminal vesicle (mg/kg bw)	64.31 ± 27.89	3799.2 ± 357.2*	94.83 ± 50.00	58.78 ± 17.67	54.06 ± 33.56
Penis (g/kg bw)	0.412 ± 0.077	0.656 ± 0.098*	0.409 ± 0.056	0.352 ± 0.05	0.298 ± 0.037*
*M. levator ani* (mg/kg bw)	668.5 ± 112.2	2471.8 ± 334.1*	649.99 ± 10.81	572.9 ± 98.2	535.5 ± 69.1*
*M. soleus* (mg/kg bw)	282.7 ± 45.66	305.2 ± 43.58	263.0 ± 25.38	220.2 ± 63.51	193.3 ± 68.38*
*M. gastrocnemius* (g/kg bw)	4.817 ± 0.416	5.263 ± 1.007	3.981 ± 0.958	4.352 ± 0.842	3.266 ± 1.398*
Liver (g/kg bw)	35.18 ± 1.50	38.77 ± 3.33	36.39 ± 1.28	36.93 ± 1.76	36.79 ± 2.89
Body weight (g)	286.5 ± 8.8	311.8 ± 21.2*	289.05 ± 19.9	287.6 ± 17.0	289.6 ± 17.3

* *p* < 0 05 denotes statistical significance compared to the orx control group.

**FIGURE 3:**
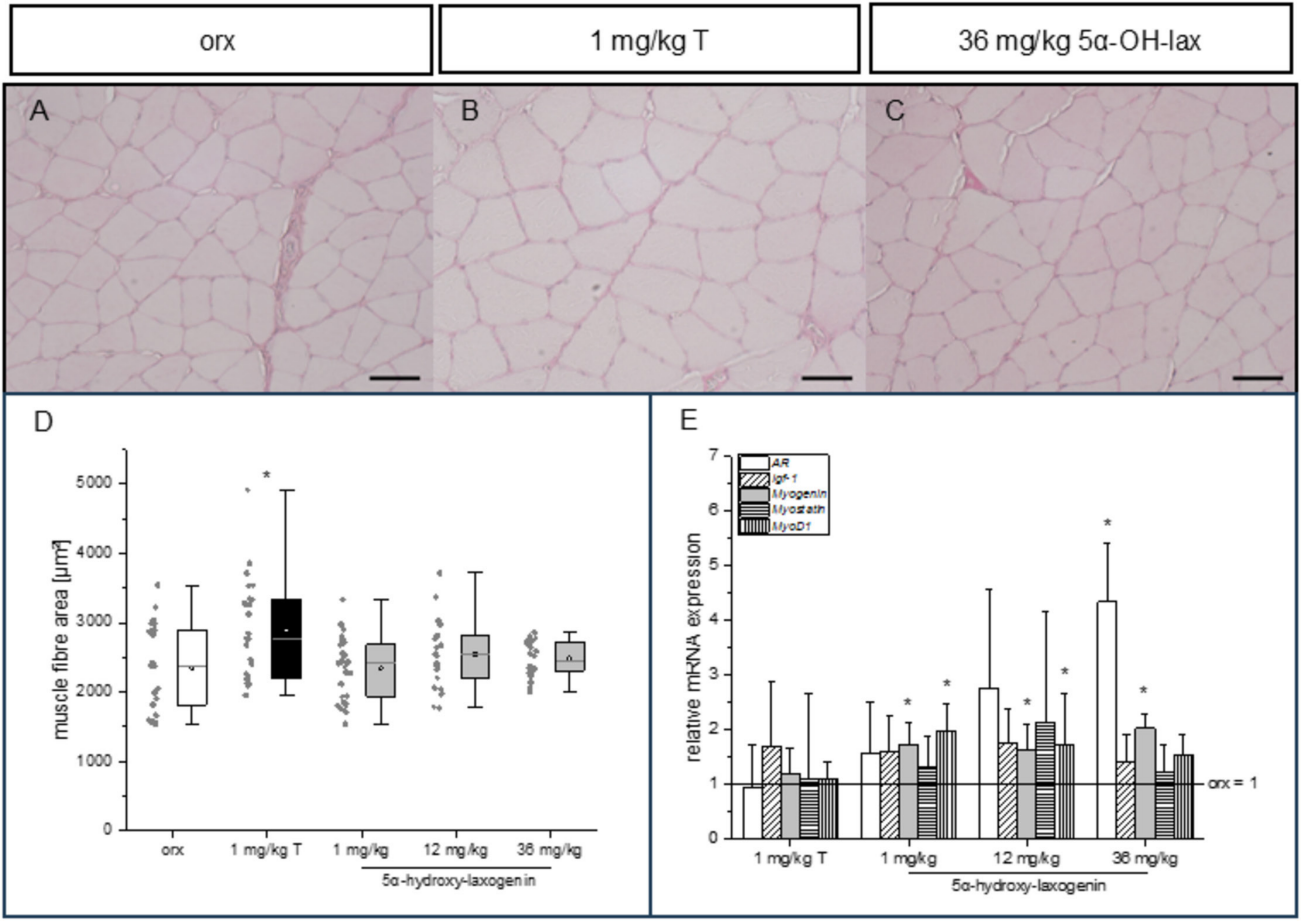
Light microscopy of PAS‐stained *M. soleus* cross sections in (A) the control group (orx), (B) the testosterone‐treated group (1 mg/kg T) and (C) the 36 mg/kg 5α‐hydroxy‐laxogenin‐treated group (36 mg/kg 5α‐OH‐lax). (D) Muscle fibre area (100/animal for 5 animals/group). Scale bar = 50 μM. *p ≤ 0.05 versus orx. (E) *M. soleus* mRNA levels relative to two reference genes and to the orx control croup (orx = 1), *p ≤ 0.05 versus orx.

An anabolic effect of the 2 weeks of testosterone substitution was observed by a significant increase in relative *musculus levator ani* wet weight (668.5 ± 112.2 mg/kg compared to 535.5 ± 69.1 mg/kg in the orx control group, Table 2). A dose‐dependent decrease of the *M. levator ani* wet weight was observed in response to the 5α‐hydroxy‐laxogenin treatment, which was statistically significant at the highest dosages of 36 mg/k (Table 2). In the two skeletal muscles investigated, testosterone treatment had no significant impact on the respective relative wet weights. In contrast, 5α‐hydroxy‐laxogenin induced a decrease of the relative wet weights of *M. soleus* und *M. gastrocnemius*, which was statistically significant in the highest dosage (Table 2). The wet weights of both skeletal muscles in the 36 mg/kg 5α‐hydroxy‐laxogenin group were only 68% of that determined in the orx control animals respectively (Table 2). Despite the lower wet weights, the cross‐sectional area of the *M. soleus* fibres was comparable among the orx control group and the 5α‐hydroxy‐laxogenin treated groups (Figure 3A,C,D). A significant increase in the muscle fibre area was only observed in the testosterone‐treated animals (Figure 3B,D). This observation is in accordance with a study by Borst et al. who also observed a significant increase in the muscle cross‐sectional area with unchanged *M. soleus* wet weights after a 30‐day treatment with 1 mg/kg testosterone [24]. The increased atrophy of *M. levator ani*, penis and the two skeletal muscles in response to the highest 5α‐hydroxy‐laxogenin dosage points to antiandrogenic or antianabolic effects respectively. However, to conclusively prove that the anabolic effect advertised by respective dietary supplements is wrong, a different setup using castrated testosterone‐substituted rats that are cotreated with 5α‐hydroxy‐laxogenin according to the OECD guideline is indicated. As experimental setup, either intact animals or gonadectomised and androgen‐substituted animals according to the OECD guideline could be used [12]. On molecular level, we quantified the mRNA levels of the AR and the myogenic regulatory factors MyoD1 and myogenin as well as myostatin, a negative regulator of myogenesis [25, 26]. Compared to the orx control group, no significant impact of the testosterone treatment was observed on the mRNA levels of the different targets in the *M. soleus* (Figure 3E). Our observation that orchiectomy does not alter the skeletal muscle *AR* gene expression is also in accordance with other studies [27, 28]. Only the *Igf‐1* mRNA level was nonsignificantly increased, 1.7‐fold compared to the control group. Except for Igf‐1, our data are in accordance with a study by Gentile et al., in which *MyoD*, *myogenin* and *myostatin* expression in the *M. soleus* of castrated rats was unaffected by DHT treatment [29]. The same was described for *MyoD* in the *M. gastrocnemius* of orchiectomized mice treated for 10 weeks with a supraphysiological testosterone dose [30]. However, in that study, the supraphysiological dosage significantly decreased myogenin and AR mRNA levels. In our study, the lack of effects might be due to the physiological testosterone dosage. In response to 5α‐hydroxy‐laxogenin treatment, the AR gene expression in the skeletal muscle was significantly upregulated in a dose‐dependent manner (Figure 3E). This upregulation points to an antiandrogenic effect as it has been described that steroid receptors are downregulated in response to an agonist [30].

One explanation for the divergent results of the in vitro [11] and the in vivo models may be dosage. In the present study, the dosages administered to the rats were chosen based on those recommended by the manufacturer of respective dietary supplements. The second highest dose administered to the rats (12 mg/kg bw) is equivalent to a daily human dose of 2 mg/kg bw (for a 75 kg individual), which in turn corresponds to the daily intake of 150 mg 5α‐hydroxy‐laxogenin advertised by dietary supplement suppliers. In the in vitro bioassay using a human prostate cell line, an AR‐dependent reporter gene expression was observed after treating the cells with 25 mg/mL and 50 mg/mL 5α‐hydroxy‐laxogenin [11]. It has been shown that the correlation between in vitro assays and especially the Hershberger assay is rather weak due to the higher physiological androgen levels necessary for AR activation [31, 32, 33]. Beside the possibility that the administered dosages were too low to activate the AR, the fast biotransformation of 5α‐hydroxy‐laxogenin into potentially inactive metabolites may also explain the lack of effects in the androgen responsive tissues [31]. This might also explain the discrepancies between the AR binding and activation in the in silico docking and in the in vitro bioassay and the lack of androgenic effects of 5α‐hydroxy‐laxogenin in the preclinical animal model. In contrast to the biotransformation in the in vivo model, the PC3(AR)_2_ cells lack expression of relevant enzymes.

## 4 Conclusion

The previously observed AR activating potency of the dietary supplement ingredient 5α‐hydroxy‐laxogenin in an in vitro bioassay was supported by the molecular docking to the human AR. However, the androgenic activity was not confirmed in the preclinical castrated rat model. 5α‐Hydroxy‐laxogenin induced neither androgenic nor anabolic effects after 2 weeks of a subcutaneous administration regime. This lack of activity might be explained by rapid degradation of 5α‐hydroxy‐laxogenin. These data dissent anabolic effects attributed to 5α‐hydroxy‐laxogenin by dietary supplement producers.

## Conflicts of Interest

The authors declare no conflicts of interest.

## Data Availability

The data that support the findings of this study are available from the corresponding author upon reasonable request.

## References

[1] V. R. Kozhuharov , K. Ivanov , and S. Ivanova , “Dietary Supplements as Source of Unintentional Doping,” BioMed Research International 2022 (2022): 8387271. 35496041 10.1155/2022/8387271 PMC9054437

[2] C. L. Torres , F. A. G. de Oliveira , L. F. Jooris , M. C. Padilha , and H. M. G. Pereira , “The Presence of Doping Agents in Dietary Supplements: A Glimpse Into the Brazilian Situation,” Drug Testing and Analysis 16 (2023): 38–48. 37161689 10.1002/dta.3517

[3] J. Zapata‐Linares and G. Gervasini , “Contaminants in Dietary Supplements: Toxicity, Doping Risk, and Current Regulation,” International Journal of Sport Nutrition and Exercise Metabolism 34, no. 4 (2024): 232–241. 38653450 10.1123/ijsnem.2023-0263

[4] Agency WA‐D , “The Prohibited List ‐ World Anti‐Doping Code, International Standard,” in *Agency WA‐D*, ed (2024).

[5] K. Walpurgis , A. Thomas , H. Geyer , U. Mareck , and M. Thevis , “Dietary Supplement and Food Contaminations and Their Implications for Doping Controls,” Food 9, no. 8 (2020): 1012. 10.3390/foods9081012 PMC7466328 32727139

[6] ”Information on Select Dietary Supplement Ingredients and Other Substances,” (2024), https://www.fda.gov/food/dietary‐supplements/information‐select‐dietary‐supplement‐ingredients‐and‐other‐substances.

[7] B. Avula , A. G. Chittiboyina , J. Y. Bae , et al., “The Power of Hyphenated Chromatography‐Time of Flight Mass Spectrometry for Unequivocal Identification of Spirostanes in Bodybuilding Dietary Supplements,” Journal of Pharmaceutical and Biomedical Analysis 167 (2019): 74–82. 30753977 10.1016/j.jpba.2018.12.045

[8] P. A. Cohen , J. Sharfstein , A. Kamugisha , and C. Vanhee , “Analysis of Ingredients of Supplements in the National Institutes of Health Supplement Database Marketed as Containing a Novel Alternative to Anabolic Steroids,” JAMA Network Open 3, no. 4 (2020): e202818. 32293681 10.1001/jamanetworkopen.2020.2818 PMC7160690

[9] Illegal Ingredient, 5‐Alpha‐Hydroxy‐Laxogenin, Appearing in More Supplements [Press Release],” USADA 25 (2022): 05.

[10] Agency WA‐D , “World Anti‐Doping Code,” in *WADA*, ed (2021).

[11] C. Beer and A. M. Keiler , “Androgenic Properties of the Dietary Supplement 5α‐Hydroxy‐Laxogenin,” Archives of Toxicology 96, no. 7 (2022): 2139–2142. 35344071 10.1007/s00204-022-03283-5 PMC9151512

[12] OECD , “OECD Guideline for the Testing of Chemicals ‐ Hershberger Bioassay in Rats: A Short‐Term Screening Assay for (Anti)Androgenic Properties,” in *OECD*, ed (2009).

[13] H. M. Berman , J. Westbrook , Z. Feng , et al., “The Protein Data Bank,” Nucleic Acids Research 28, no. 1 (2000): 235–242. 10592235 10.1093/nar/28.1.235 PMC102472

[14] P. C. Hawkins , A. G. Skillman , G. L. Warren , B. A. Ellingson , and M. T. Stahl , “Conformer Generation With OMEGA: Algorithm and Validation Using High Quality Structures From the Protein Databank and Cambridge Structural Database,” Journal of Chemical Information and Modeling 50, no. 4 (2010): 572–584. 20235588 10.1021/ci100031x PMC2859685

[15] P. C. Hawkins , A. G. Skillman , and A. Nicholls , “Comparison of Shape‐Matching and Docking as Virtual Screening Tools,” Journal of Medicinal Chemistry 50, no. 1 (2007): 74–82. 17201411 10.1021/jm0603365

[16] L. Cantin , F. Faucher , J. F. Couture , et al., “Structural Characterization of the Human Androgen Receptor Ligand‐Binding Domain Complexed With EM5744, a Rationally Designed Steroidal Ligand Bearing a Bulky Chain Directed Toward Helix 12,” Journal of Biological Chemistry 282, no. 42 (2007): 30910–30919. 17711855 10.1074/jbc.M705524200

[17] Molecular Operating Environment (MOE) , Chemical Computing Group ULC: 1010 Sherbooke St. West, (Montreal, QC, Canada: Suite #10, 2020).

[18] P. Labute , “Protonate3D: Assignment of Ionization States and Hydrogen Coordinates to Macromolecular Structures,” Proteins 75, no. 1 (2009): 187–205. 18814299 10.1002/prot.22234 PMC3056144

[19] K. Pereira de Jesus‐Tran , P. L. Cote , L. Cantin , J. Blanchet , F. Labrie , and R. Breton , “Comparison of Crystal Structures of Human Androgen Receptor Ligand‐Binding Domain Complexed With Various Agonists Reveals Molecular Determinants Responsible for Binding Affinity,” Protein Science 15, no. 5 (2006): 987–999. 16641486 10.1110/ps.051905906 PMC2242507

[20] G. Jones , P. Willett , R. C. Glen , A. R. Leach , and R. Taylor , “Development and Validation of a Genetic Algorithm for Flexible Docking,” Journal of Molecular Biology 267, no. 3 (1997): 727–748. 9126849 10.1006/jmbi.1996.0897

[21] T. A. Halgren and R. B. Nachbar , “Merck Molecular Force Field. IV. Conformational Energies and Geometries for MMFF94,” Journal of Computational Chemistry 17 (1996): 587–615.

[22] G. Wolber and T. Langer , “LigandScout: 3‐D Pharmacophores Derived From Protein‐Bound Ligands and Their Use as Virtual Screening Filters,” Journal of Chemical Information and Modeling 45, no. 1 (2005): 160–169. 15667141 10.1021/ci049885e

[23] X. E. Zhou , K. M. Suino‐Powell , J. Li , et al., “Identification of SRC3/AIB1 as a Preferred Coactivator for Hormone‐Activated Androgen Receptor,” Journal of Biological Chemistry 285, no. 12 (2010): 9161–9171. 20086010 10.1074/jbc.M109.085779 PMC2838335

[24] S. E. Borst , C. F. Conover , C. S. Carter , et al., “Anabolic Effects of Testosterone Are Preserved During Inhibition of 5Alpha‐Reductase,” American Journal of Physiology. Endocrinology and Metabolism 293, no. 2 (2007): E507–E514. 17488806 10.1152/ajpendo.00130.2007

[25] C. Vicente‐Garcia , J. D. Hernandez‐Camacho , and J. J. Carvajal , “Regulation of Myogenic Gene Expression,” Experimental Cell Research 419, no. 1 (2022): 113299. 35926660 10.1016/j.yexcr.2022.113299

[26] S. J. Lee , “Myostatin: A Skeletal Muscle Chalone,” Annual Review of Physiology 85 (2023): 269–291. 10.1146/annurev-physiol-012422-112116 PMC10163667 36266260

[27] P. J. Roch , V. Wolgast , M. M. Gebhardt , et al., “Combination of Selective Androgen and Estrogen Receptor Modulators in Orchiectomized Rats,” Journal of Endocrinological Investigation 45, no. 8 (2022): 1555–1568. 35429299 10.1007/s40618-022-01794-7 PMC9270269

[28] J. Antonio , J. D. Wilson , and F. W. George , “Effects of Castration and Androgen Treatment on Androgen‐Receptor Levels in Rat Skeletal Muscles,” Journal of Applied Physiology 87, no. 6 (1985): 2016–2019. 10.1152/jappl.1999.87.6.2016 10601143

[29] M. A. Gentile , P. V. Nantermet , R. L. Vogel , et al., “Androgen‐Mediated Improvement of Body Composition and Muscle Function Involves a Novel Early Transcriptional Program Including IGF1, Mechano Growth Factor, and Induction of Beta‐Catenin,” Journal of Molecular Endocrinology 44, no. 1 (2010): 55–73. 19726620 10.1677/JME-09-0048

[30] K. Rana , N. K. Lee , J. D. Zajac , and H. E. MacLean , “Expression of Androgen Receptor Target Genes in Skeletal Muscle,” Asian Journal of Andrology 16, no. 5 (2014): 675–683. 24713826 10.4103/1008-682X.122861 PMC4215656

[31] E. Sonneveld , J. A. Riteco , H. J. Jansen , et al., “Comparison of In Vitro and In Vivo Screening Models for Androgenic and Estrogenic Activities,” Toxicological Sciences 89, no. 1 (2006): 173–187. 16221957 10.1093/toxsci/kfj009

[32] W. G. Schoonen , G. H. Deckers , M. E. de Gooijer , R. de Ries , and H. J. Kloosterboer , “Hormonal Properties of Norethisterone, 7Alpha‐Methyl‐Norethisterone and Their Derivatives,” Journal of Steroid Biochemistry and Molecular Biology 74, no. 4 (2000): 213–222. 11162927 10.1016/s0960-0760(00)00125-4

[33] W. G. Schoonen , G. Deckers , M. E. de Gooijer , et al., “Contraceptive Progestins. Various 11‐Substituents Combined With Four 17‐Substituents: 17Alpha‐Ethynyl, Five‐ and Six‐Membered Spiromethylene Ethers or Six‐Membered Spiromethylene Lactones,” Journal of Steroid Biochemistry and Molecular Biology 74, no. 3 (2000): 109–123. 11086230 10.1016/s0960-0760(00)00094-7

